# Differential baseline and response profile to IFN-γ gene transduction of IL-6/IL-6 receptor-α secretion discriminate primary tumors versus bone marrow metastases of nasopharyngeal carcinomas in culture

**DOI:** 10.1186/1471-2407-9-169

**Published:** 2009-06-05

**Authors:** Andy Shau-Bin Chou, Hsin-Yi Wang, Hung-Chang Chen, Ming-Hsiu Tsai, Cheng-Keng Chuang, Shuen-Kuei Liao

**Affiliations:** 1 Graduate Institute of Clinical Medical Sciences, College of Medicine, Chang Gung University, Taoyuan, Taiwan; 2 Graduate Institute of Biomedical Sciences, College of Medicine, Chang Gung University, Taoyuan, Taiwan; 3 Department of Otolaryngology, China Medical University Hospital, Taichung, Taiwan; 4 Division of Urology, Department of Surgery, Chang Gung Memorial Hospital, Taoyuan, Taiwan; 5 Department of Radiology, Tzu-Chi General Hospital, Hualien, Taiwan

## Abstract

**Background:**

Understanding of immunobiology of bone marrow metastases (designated BM-NPC) *versus *primary tumors (P-NPC) of the nasopharynx is far from complete. The aim of this study was to determine if there would be differences between cultured P-NPCs and BM-NPCs with respect to (i) constitutive IL-6 and the IL-6 receptor gp80 subunit (IL-6Rα) levels in the spent media of nontransduced cells, and (ii) IL-6 and IL-6Rα levels in the spent media of cells transduced with a retroviral vector containing the *IFN-γ *gene.

**Methods:**

A panel of NPC cell lines were transduced with the *IFN-γ *gene through a retroviral vector. Four clonal sublines were isolated *via *limiting dilution methods. Cytofluorometric analysis was performed for the detection of cell surface antigens of HLA class I, HLA class II and ICAM-1. ELISA was used to assay for IFN-γ, IL-6 and IL-6Rα in the spent media of cultured cell lines.

**Results:**

Our results showed that in day 3 culture supernatants, low levels of soluble IL-6 were detected in 5/5 cultured tumors derived from P-NPCs, while much higher constitutive levels of IL-6 were detected in 3/3 metastasis-derived NPC cell lines including one originated from ascites; the difference was significant (*p *= 0.025). An inverse relationship was found between IL-6Rα and IL-6 in their release levels in cultured P-NPCs and metastasis-derived NPCs. In *IFN-γ*-transduced-P-NPCs, IL-6 production increased and yet IL-6Rα decreased substantially, as compared to nontransduced counterparts. At variance with P-NPC cells, the respective ongoing IL-6 and IL-6Rα release patterns of BM-NPC cells were not impeded as much following *IFN-γ *transduction. These observations were confirmed by extended kinetic studies with representative NPC cell lines and clonal sublines. The latter observation with the clonal sublines also indicates that selection for high IL-6 or low IL-6Rα producing subpopulations did not occur as a result of *IFN-γ*-transduction process. P-NPCs, which secreted constitutively only marginal levels of IFN-γ (8.4 ~ 10.5 pg/ml), could be enhanced to produce higher levels of IFN-γ (6.8- to 10.3-fold increase) after *IFN-γ *transduction. Unlike P-NPCs, BM-NPCs spontaneously released IFN-γ at moderate levels (83.8 ~ 100.7 pg/ml), which were enhanced by 1.3- to 2.2-fold in the spent media of their *IFN-γ*-transduced counterparts.

**Conclusion:**

Our results showed that cultured P-NPCs and BM-NPCs could be distinguished from one another on the basis of their differential baseline secretion pattern of IFN-γ, IL-6 and IL-6Rα, and their differential response profiles to *IFN-γ *gene transfer of the production of these three soluble molecules. These results suggest that the IL-6 and IFN-γ pathways in a background of genetic instability be involved in the acquisition of metastatic behaviour in BM-NPCs.

## Background

Interferon-γ (IFN-γ) is known to function as a factor of growth, differentiation or activation for a variety of immune cells, such as macrophages, natural killer (NK) cells and cytotoxic T lymphocytes (CTLs) at the site of tumor in patients [1]. Furthermore, IFN-γ induces enhanced expression of key immune regulatory molecules such as class I and class II major histocompatibility complex (MHC) molecules and intercellular adhesion molecule-1 (ICAM-1) on the surface of tumor cells, which may lead to improved antigen presentation to dendritic cells as well as to pre-existing primed CTLs for further expansion and enhanced destruction of tumor cells [2,3]. One way toward this aim is to assure the continuous presence of IFN-γ locally in the tumor lesion by introducing the *IFN-γ *gene into the tumor cells constitutively [2-5]. The resultant IFN-γ production at the tumor site should specifically increase levels of class I and class II HLA as well as ICAM-1 expression on the tumor cells. Over the past two decades, increasing numbers of tumor rejection antigens (processed peptides) presented by specific MHC class I (HLA class I) molecules recognized by human T cells have been identified not only in malignant melanoma but also in a variety of other solid tumors including nasopharyngeal carcinoma (NPC) [6-8].

Human NPC, which is universally associated with Epstein-Barr virus (EBV), is a tumor of epithelial origin with endemic distribution among certain well-defined ethnic groups and geographic regions [9,10]. The highest incidence of the disease (25–50/100,000 persons per year, accounting for approximately 30% of all cancers) has been observed in southern Chinese, who live in Guangdong and Guangxi provinces as well as in a certain part of Taiwan. NPC patients with stage I and II are usually treated with radiotherapy alone and have a 5-year survival rate of greater than 75%. However, a 5-year survival rate of NPC patients with stage III or IV after radiotherapy vary considerably, ranging from 18 ~ 52%, in spite of improved results with combined use of radiotherapy and chemotherapy. Patients with metastatic disease diagnosed at the beginning of therapy almost all die within 4 years. Thus, a more effective mode of therapy for NPC is urgently needed. While most currently available NPC cell lines are of local, primary tumor origin [11-14], cell lines derived from metastatic sites are rarely available to be studied for the understanding of the natural history and immunotherapy of this malignancy [15]. Since bone marrow is one of major common metastatic sites (following bone, lung and liver) with a reported incidence of 22% [16], bone marrow provides an enduring reservoir and sanctuary, from which tumor cells could spread to other organs through the circulation. Although the importance of micrometastatic bone marrow involvement of most solid tumors in relation to poor prognosis has long been recognized, the nature of these bone marrow metastatic tumor cells has not been well defined [15,17,18]. Thus a positive bone marrow finding for NPC cells may be a selection criterion for adjuvant therapy for the patients with minimal residual tumor load. The invasive phenotype of the tumor cells in bone marrow is reflected by their epithelial-mesenchymal transition (EMT) and expression of several metastasis-associated molecules, which may be potential candidates for therapeutic targets [19,20]. We have been interested in looking into this aspect of biological questions, and therefore established two NPC cell lines from bone marrow metastatic lesions. Such cell lines established in this laboratory have been described in detail elsewhere [15,21].

During the course of our efforts toward the development of tumor vaccines for NPC by transfer of the *IFN-γ *gene to NPC cell lines *via *a retroviral vector, we noted that substantial changes occurred in the levels of IL-6 and one of its receptor subunits, gp80, commonly referred to as IL-6Rα, released by NPC cell lines after *IFN-γ *transduction. Although IL-6 is expressed and released by NPC cells to various extents, its exact role in the process of NPC progression and bone marrow micrometastasis has largely not been investigated [19]. In this study we describe the distinct differences between cultured NPCs derived from primary sites (P-NPCs) and those from bone marrow metastatic lesions (BM-NPCs) in regard to basal levels of IL-6 and IL-6Rα released constitutively and to the profiles of these two soluble molecules in response to *IFN-γ *gene transduction.

## Methods

### Cells and culture conditions

Human NPC cell lines used were the following: NPC039 and NPC076, which both derived from NPC primary sites [12] were gifts from Dr. C-T Lin, Department of Pathology, National Taiwan University, Taipei, Taiwan; two additional cell lines, CNE-1 and CNE-2 [11], originally established from NPC primary lesions in the laboratory of Dr. Y Zeng (Chinese Academy of Preventive Medicine, Beijing, China) were kindly supplied to us through Dr. K-C. Chow, China Medical University, Taichung, Taiwan, with the permission from Dr. Y Zeng; and NPC-BR, a cell line derived from a NPC primary site, two additional cell lines NPC-BM1 [15,21] and NPC-BM2 [21], derived from bone marrow metastatic lesions, were established in this laboratory; CNE-3, also established by Dr. Y Zeng from malignant ascites of a NPC patient in China, was a gift from Dr. Y Zeng. Four clonal sublines, NPC039-2E2, -6A1, -7B2 and -9A2 were isolated from the NPC039 cell line through a limiting dilution method and the isolates were grown and maintained in culture. NPC-BM3, a short-term culture derived from a bone marrow metastatic lesion from the third patient with NPC was also included in this study.

The culture medium used was RPMI-1640 (GIBCO, Grand Island, NY) supplemented with 1 mM sodium pyruvate, 2 mM glutamine, 100 U/ml penicillin-G, 100 μg/ml streptomycin sulfate, and 10% fetal bovine serum (FBS). Cultures were maintained at 37°C in an incubator with 5% CO_2_, 95% air and humidified atmosphere. In the routine maintenance of our cell lines, medium was changed every 3 – 4 days. When growth reached near confluence, cells were subcultured. Intermittently, some cells were cryopreserved in RPMI-1640 medium containing 10% FBS and 10% dimethyl sulfoxide in liquid nitrogen.

### Monoclonal antibodies

The monoclonal antibody (mAb W6/32) to a monomorphic determinant of HLA class I molecules associated with β_2_-microglobulin was purchased from DAKO Japan Co., Ltd., Kyoto, Japan. Both mAb to HLA class II (clone DK22, specific for the β-chain of all DR loci, all DP loci, DQw1 but not with DQw3 locus) and mAb to ICAM-1 (CD54) (clone 6.5B5) were purchased from DakoCytomation, Glostrup, Denmark.

### Retroviral vectors

A vector containing the gene for human *IFN-γ*, the neomycin phosphotransferase (*neo*) gene conveying resistance to neomycin, and the *LacZ *gene encoding for *Escherichia coli *β-galactosidase (β-Gal) was provided by Chiron Viagene (San Diego, CA). The properties of this *IFN-γ *vector have been previously described [5]. Briefly, it carried the *IFN-γ *gene under the control of the murine retrovirus (Moloney murine leukemia virus, M-MLV) long terminal repeat and the simian virus (SV40) early promoter driving the neogene. The vector was introduced to the dog cell (D17) amphotropic packaging line known as DA. The clonal producer line DA/IFN-γ was isolated and used to produce a vector preparation with a titer of 5.6 × 10^6^ colony-forming units (cfu) per ml when tested on human HT-1080 cells. A similar vector, *CBβ-Gal*, instead of *IFN-γ *was used to prepare the clonal producer line DA/CBβ-Gal, which produced a vector preparation with a titer of approximately 5.5 × 10^6 ^cfu/ml on HT-1080 cells. Additionally, a neovector without containing the *IFN-γ *gene was used as a control. The quantity of vectors used for transduction of each cell line or subline was expressed as a multiplicity of infection (MOI).

### Optimization of transduction conditions

A retroviral vector containing a marker gene for the enzyme β-Gal was first transduced in order to optimize the efficiency of transduction using cytochemical methods. In a checkerboard titration manner, tumor cells (1 × 10^6^) were cultured in different sets of T-75 flasks in complete growth medium containing polybrene (Sigma Chemicals, St. Louis, MO) (0.5 ~ 16 μg/ml) and *β*-*Gal *vector (1 ~ 20 cfu/cell) at different concentrations. Three days later, transduced monolayer tumor cells were washed in PBS, fixed with 1% formaldehyde, and stained with X-Gal. At least 200 cells were counted to determine transduction efficiency by the percentage of positively stained cells exhibiting uniformly blue cytoplasm.

### Retroviral transduction of the *IFN*-*γ* gene

Tumor cells (1 × 10^6^) of a given tumor cell line, subline, or culture were allowed to grow overnight in T-75 flasks with RPMI-1640 medium containing 10% FBS, polybrene at predetermined concentration and the *IFN-γ *vector at predetermined (preoptimized) MOI. The following day, G418 at the predetermined minimal concentration that was completely lethal to neomycin-sensitive cells was added to initiate the selection of neomycin-resistant (i.e. transduced) cells by elimination of nontransduced cells with that concentration of G418. After complete of the selection, medium in flasks containing attached transduced cells was replaced with fresh complete growth medium and changed every 3 ~ 4 days thereafter. The properties of these cell lines and clonal sublines with respect to their origins of derivation (primary *versus *metastatic lesions) and success or failure in transduction with the retroviral vector containing *IFN-γ *are listed in Table 1.

**Table 1:** Sensitivity levels to G418 killing and success/failure in *IFN-γ*-transduction of a panel of NPC cell lines/sublines

Cell line/clonal subline	Sensitivity to G418 killing (minimal dose for 100% cell kill) (μg/ml)	Success in *IFN-γ *gene transduction *via* a retroviral vector
***Derived from a primary lesion***		
		
NPC039	600	Yes
NPC076	600	Yes
NPC-BR	300	Yes
CNE-1	600	No
CNE-2	600	No
NPC039-2E2*	600	Yes
NPC039-6A1*	600	Yes
NPC039-7B2*	600	Yes
NPC039-9A2*	600	Yes
		
***Derived from a metastatic lesion***		
		
CNE-3 (ascites)	18,000	No
NPC-AS1 (ascites)	9,000	No
NPC-BM1 (bm^§^)	18,000	Yes
NPC-BM2 (bm)	12,000	No
NPC-BM3 (bm)	15,000	Yes

*These four sublines were clonally isolated from the NPC039 cell line.

^§^The abbreviation means that the cell line or culture was derived from a bone marrow metastatic lesion.

### Cytofluorometric analysis

Monodispersed cells harvested from monolayer cultures by trypsinization were distributed into test tubes (5 × 10^5 ^cells/tube) and centrifuged at 400 × g to pellet the cells. Test antibody samples (5 μg/ml; 100 μl/tube) were added to the cells, and the mixtures were incubated at 4°C for 30 min. Bound antibodies were detected by fluoresencein-isothiocyanate (FITC)-conjugated polyclonal antibodies (goat-anti-mouse IgG; Coulter Immunology, Hialeah, FL) in flow cytometry according to the procedures described previously [22]. Appropriate positive and negative control antibodies, such as anti-HLA class I (mAb W6/32), phosphate-buffered saline (PBS) and isotype-matched irrelevant mAbs were included in each experiment.

### Enzyme-linked immunosorbent assay (ELISA)

ELISA kits for quantification of human IFN-γ, IL-6 and IL-6Rα were all purchased from R&D Systems, Inc., Minneapolis, MN. The assays were carried out according to the manufacturer's instructions. The limits of detectable levels of IFN-γ, IL-6 and IL-6Rα assays were 8.0, 0.7 and 3.5 pg/ml, respectively. The primary mAbs used in the assays were neutralizing antibodies and therefore the cytokines detected only bioactive IFN-γ, IL-6 and IL-6Rα. No IL-6 and IL-6Rα was detected in the fresh medium used in culture. Furthermore, we have performed experiments to confirm the notion from the manufacture that the presence of IL-6Rα or IL-6 in the medium does not interfere with the measurements of IL-6 or IL-6Rα, respectively, with their assay kits.

### Statistical analysis

Day 3 spent media of 8 NPC cell lines (five from primary lesions and three from metastatic lesions) were collected for measurements of their IL-6 and IL-6Rα secretion. The simple linear regression analysis was performed to determine the correlation between IL-6 and logarithmic transformed IL-6Rα secretion in the eight NPC cell lines. These data were further analyzed by using of Mann-Whitney U test to compare the secretion of IL-6 and IL-6Rα between primary and metastatic cell lines each as a group. The *p *values calculated to be less than 0.05 were considered to indicate statistically differences between the primary and metastatic groups. All statistic analyses were performed with the SPSS version 13.0 (SPSS Inc., Chicago, Ill).

## Results

### Phenotypic characterization of the NPC cell lines

A panel of 14 NPC cell lines/sublines were tested initially, which included 5 cell lines derived from primary lesions (NPC039, NPC076, NPC-BR, CNE-1, CNE-2), 4 clonal sublines derived from the NPC039 cell line (NPC039-2E2, -6A1, -7B2, -9A2), and 5 cell lines from metastatic lesions (CNE-3 and NPC-AS1 from ascites; NPC-BM1, -BM2, and -BM3, the latter being a short-term culture from different bone marrow lesions). Apart from the differences in the sensitivity to G418 killing, susceptibility to *IFN-γ *transduction, these were different from one another with respect to the extent of upregulation of HLA class I, HLA class II and ICAM-1 expression in response to *IFN-γ *transduction (Table 2). Since greater than 90% of cells in the cell lines tested were positive for HLA class I, the relative mean fluorescence intensity (MFI) indicative of antigenic density on the cell surface was used to compare the differences between nontransduced and transduced cells of a given NPC cell line. These results illustrate the heterogeneity and individuality of these NPC cell lines examined, and in all instances where pairs of transduced and nontransduced cells were available for direct comparison. There was a 1.4 to 3.2-fold higher expression of surface HLA class I antigens by the *IFN-γ*-transduced cells than that by the nontransduced cells. It is of note that among the NPC cell lines tested, HLA class II seem to be expressed exclusively by those derived from metastatic sites, the expression of which was upregulated on *IFN-γ*-transduced cells.

**Table 2:** Expression of surface HLA class I, HLA class II and ICAM-1 by nontransduced and *IFN-γ-*transduced NPC cell lines as determined by cytofluorometric analysis

Cell line/Subline	HLA class I	HLA class II	ICAM-1
***Derived from primary lesion***			
			
**NPC039**			
Nontransduced	99.9 (73.0) *	0.4	99.7 (22.1)
Transduced	99.3 (231.8)	40.1 (22.0)	99.3 (61.1)
**NPC076**			
Nontransduced	97.9 (96.8)	0.1	93.2 (16.8)
Transduced	94.1 (147.3)	28.4 (30.8)	90.8 (44.7)
**NPC-BR**			
Nontransduced	95.8 (78.7)	0.5	95.9 (25.4)
Transduced	97.9 (139.7)	48.7 (36.2)	96.7 (50.2)
**NPC039-2E2^§^**			
Nontransduced	99.9 (138.8)	0.1	86.9 (13.5)
Transduced	99.9 (179.5)	72.4 (44.0)	96.6 (30.5)
**NPC039-6A1^§^**			
Nontransduced	89.5 (71.6)	2.3 (8.2)	79.1 (13.6)
Transduced	97.6 (283.0)	48.8 (47.7)	86.0 (27.3)
**NPC039-7B2^§^**			
Nontransduced	99.6 (44.3)	0.9 (6.8)	90.7 (10.2)
Transduced	98.8 (88.7)	39.5 (58.0)	95.3 (23.9)
***Derived from bone marrow metastasis***			
			
**NPC-BM1**			
Nontransduced	91.2 (55.3)	26.2 (28.8)	87.8 (26.9)
Transduced	96.8 (97.6)	41.9 (28.2)	91.7 (28.8)
**NPC-BM3**			
Nontransduced	89.4 (78.2)	18.6 (33.4)	87.1 (19.6)
Transduced	93.1 (121.6)	66.7 (50.5)	93.8 (49.2)

### Levels of IFN-γ released by *IFN*-*γ*-transduced and nontransduced NPC cell lines

**Figure 1:**
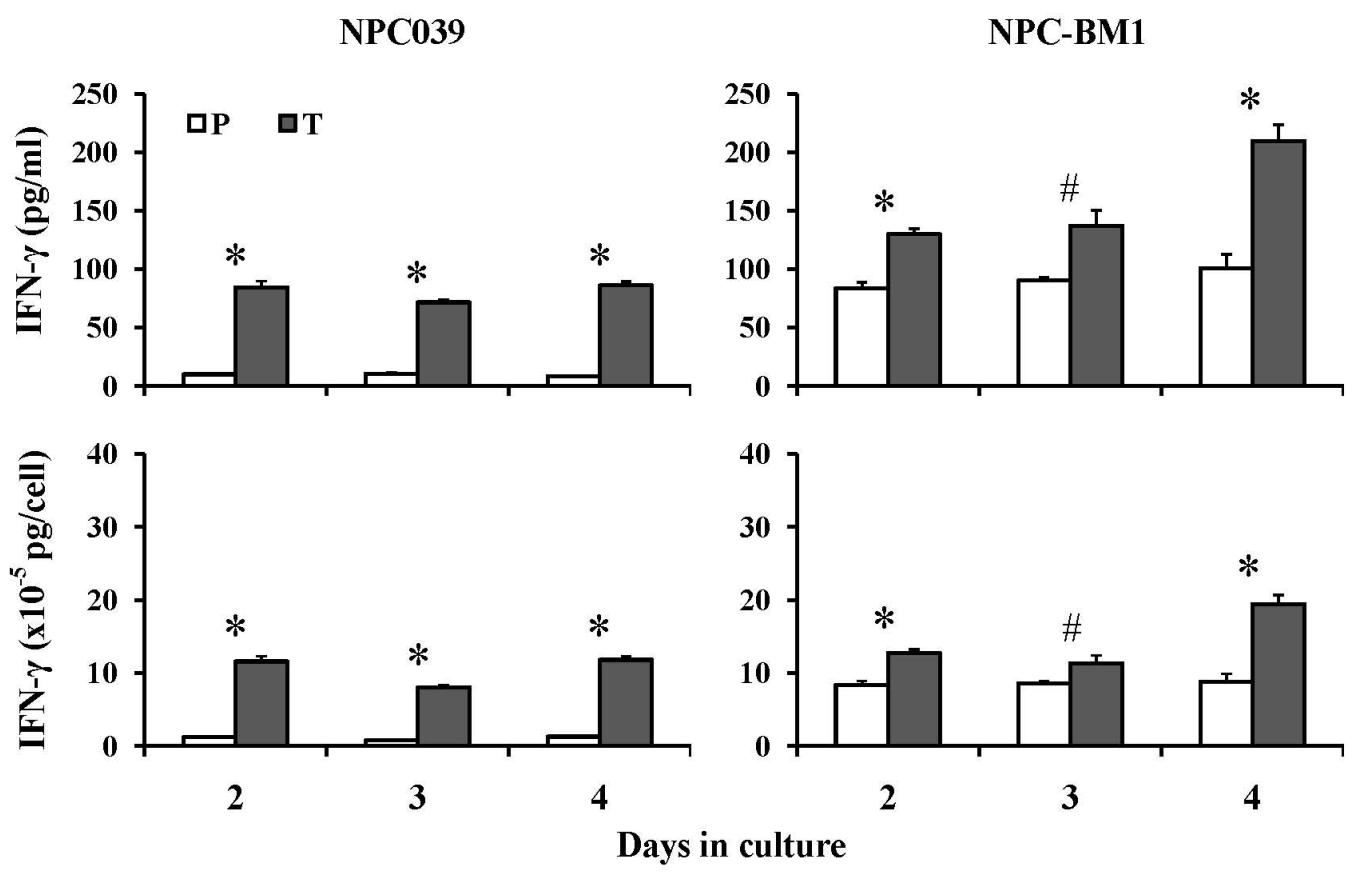
**Release of IFN-γ protein in the spent media by *IFN-γ*-transduced (T) and nontransduced parental (P) cells of the NPC039 or NPC-BM1 line**. Results of IFN-γ secreted are expressed on the basis of pg per ml and pg per cell for the NPC039 cell line (left panel) and the NPC-BM1 cell line (right panel).

The culture spent media of *IFN-γ*-transduced and nontransduced cells of the NPC039 cell line were tested for IFN-γ protein secretion at day 2, 3 and 4 by ELISA (Figure 1). There were only little or marginal levels of IFN-γ detected in the spent media of cultured nontransduced NPC039 cells at the three time points. In *IFN-γ*-transduced NPC039 cells, IFN-γ was detected at day 2 (84.5 pg/ml or 11.6 × 10^-5 ^pg/cell), which remained at the similar levels up to day 4. On the other hand, IFN-γ released into the spent media of nontransduced NPC-BM1 cells was readily detected at day 2 (83.8 pg/ml) and thereafter the level remained at the similar levels ranging from 83.8 ~ 100.7 pg/ml. When analyzed on per cell basis, its value remained at the similar level over the observation period in the range of 8.38 × 10^-5 ^~ 8.83 × 10^-5 ^pg/cell (Figure 1, right panel). In *IFN-γ*-transduced NPC-BM1 cells, the amounts of IFN-γ detected at day 2 and day 3 were comparable in the range of 129.9 ~ 137.2 pg/ml, or 11.3 × 10^-5 ^~ 12.7 × 10^-5 ^pg/cell, the level which was increased to 209.8 pg/ml, or 19.4 × 10^-5 ^pg/cell at day 4.

### Release of IL-6 in cultured NPC cell lines derived from primary and metastatic lesions

**Figure 2:**
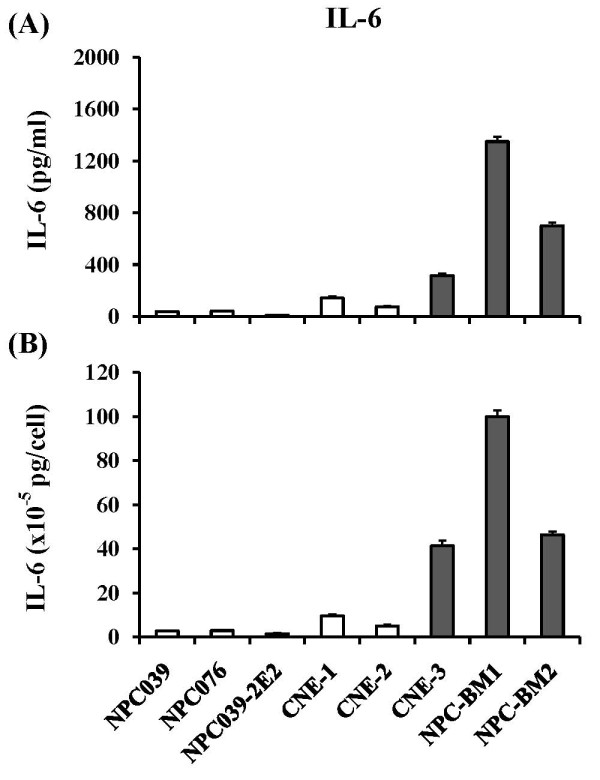
**Levels of IL-6 detected in the supernatants by nontransduced NPC cell lines at day 3 cultures**. Results of IL-6 secreted are expressed on the basis of per ml (penal A) and per cell (panel B). Note that NPC039, NPC076, NPC039-2E2, CNE-1 and CNE-2 were derived from primary NPC lesions (open columns), while CNE-3 (ascites), NPC-BM1 (bone marrow) and NPC-BM2 (bone marrow) were derived from different metastatic sites indicated (closed gray columns).

We next examined the constitutive levels of IL-6 released by eight cultured (nontransduced) NPC cell lines including those derived from 5 primary and 3 metastatic lesions. The spent media were collected and tested for soluble IL-6 at day 3 cultures of NPC cell lines. Results in Figure 2 revealed that four cultured P-NPCs (NPC039, NPC076, CNE-1, CNE-2) and one clonal sublines NPC039-2E2 released low levels of IL-6 [60.8 ± 50.7 pg/ml or (4.3 ± 3.2) × 10^-5 ^pg/cell]. On the other hand, three metastatic NPC cell lines (CNE-3, NPC-BM1, NPC-BM2) released much higher levels of IL-6 [787.7 ± 522.9 pg/ml or (62.5 ± 32.5) × 10^-5 ^pg/cell]. The difference between these two groups of NPC cell lines was statistically significant.

### Release of IL-6Rα in cultured NPC cell lines derived from primary and metastatic lesions

**Figure 3:**
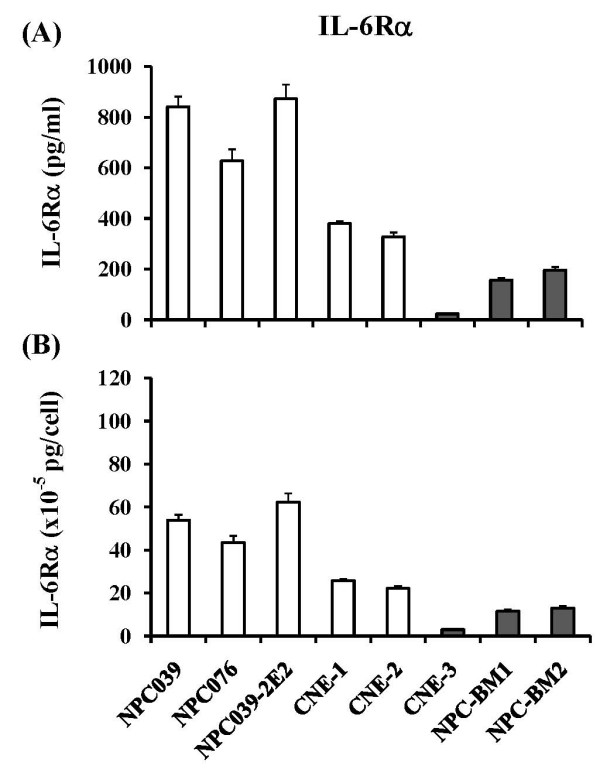
**Levels of IL-6Rα detected in the spent media by nontransduced NPC cell lines at day 3 cultures**. Results of IL-6Rα secreted are expressed on the basis of per ml (penal A) and per cell (penal B). The penal of cell lines used were same as those in Figure 2.

Experiments were set up to determine soluble IL-6Rα levels in the same day 3 culture media of the eight NPC cell lines used for the measurement of IL-6 above. Results in Figure 3A showed that 4 P-NPC cell lines and 1 subline derived from P-NPC released significantly higher amounts of IL-6Rα than 3 NPC cell lines from metastatic lesions did (609.5 ± 252.8 pg/ml *versu*s 124.6 ± 90.1 pg/ml). A significant difference in constitutive release profiles of soluble IL-6Rα between cultured NPCs from primary lesions and cultured NPCs from metastatic lesions was also evident, when the results were expressed by pg of the receptor per cell in the culture supernatant [(41.5 ± 17.4) × 10^-5 ^pg/cell *versus *(9.1 ± 5.4) × 10^-5 ^pg/cell] (Figure 3B).

### Secretion pattern differences in IL-6/IL-6Rα between cultured NPC cell lines derived from primary *versus *metastatic lesions

**Figure 4:**
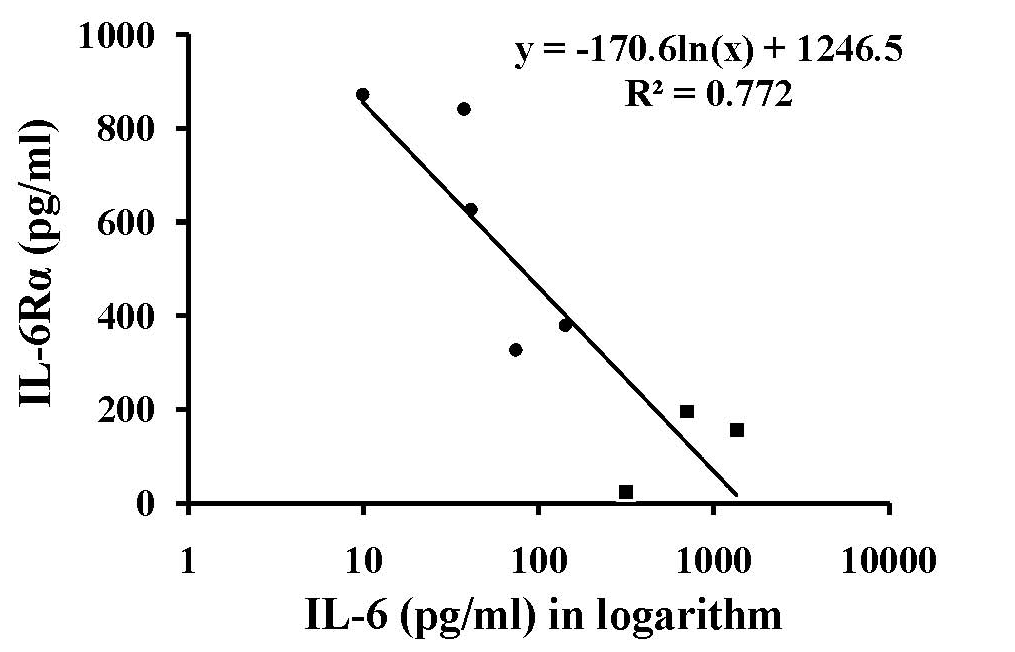
**Statistical analysis of the relationship between IL-6 and IL-6Rα secreted spontaneously by NPC cell lines derived from primary and metastatic lesions**. Regression curve constructed based on the relationship between IL-6 and IL-6Rα secreted at day 3 by all NPC cell lines tested including five P-NPCs (•) and three metastasis-derived NPC cell lines (■). The line was calculated by linear regression (r = 0.878; *p *< 0.01).

To evaluate the relationship between secretion of IL-6 and IL-6Rα in these eight NPC cell lines, the observed data of cell lines were used to construct a two-way dot plot analysis for estimating whether a simple linear regression line would fit in these two parameters. We found that as the secretion of IL-6Rα was increased, the secretion of IL-6 was decreased in an exponential manner. Thus, a logarithmic transformation of IL-6 values was made to fit a simple linear regression model. In Figure 4, a linear relationship based on this linear regression analysis is indicated, showing an inverse correlation between IL-6 and IL-6Rα (R^2 ^= 0.772, *p *< 0.01).

The distributions of IL-6 and IL-6Rα secreted by NPC cell lines derived from primary lesions (n = 5) and by NPC cell lines from metastatic lesions (n = 3) are summarized in Table 3. The amounts of IL-6 secreted by P-NPC cell lines (60.8 ± 50.7 pg/ml) were clearly much less than metastasis-derived NPC cell lines (787.7 ± 522.9 pg/ml). On the other hand, the amounts of IL-6Rα secreted by P-NPC cell lines (609.5 ± 252.8 pg/ml) were much higher than those by metastasis-derived NPC cell lines (124.6 ± 90.1 pg/ml). As analyzed by Mann-Whitney U test, the secretion pattern of IL-6 exhibited by P-NPCs was significantly different from that of metastasis-derived NPC cell lines (*p *= 0.025), so was between them with respect to the release pattern of IL-6Rα (*p *= 0.025).

**Table 3:** Comparative production of IL-6/IL-6Rα by P-NPC and metastasis-derived NPC cell lines at day 3 *in vitro *culture

Variable	P-NPC cell lines (n = 5)	Metastasis-derived NPC cell lines (n = 3)	*p *value^#^
IL-6 (pg/ml)	60.8 ± 50.7 ^†^	787.7 ± 522.9	0.025
IL-6Rα (pg/ml)	609.5 ± 252.8	124.6 ± 90.1	0.025

^† ^Results were expressed as mean ± SD. *p *values were calculated by using ^#^Mann-Whitney U test for comparison of IL-6 and IL-6Rα production in culture.

### Differential effects of *IFN*-*γ* gene transduction on the kinetic secretion of soluble IL-6 and IL-6Rα from cultured primary NPC tumors and metastatic NPC tumors

**Figure 5:**
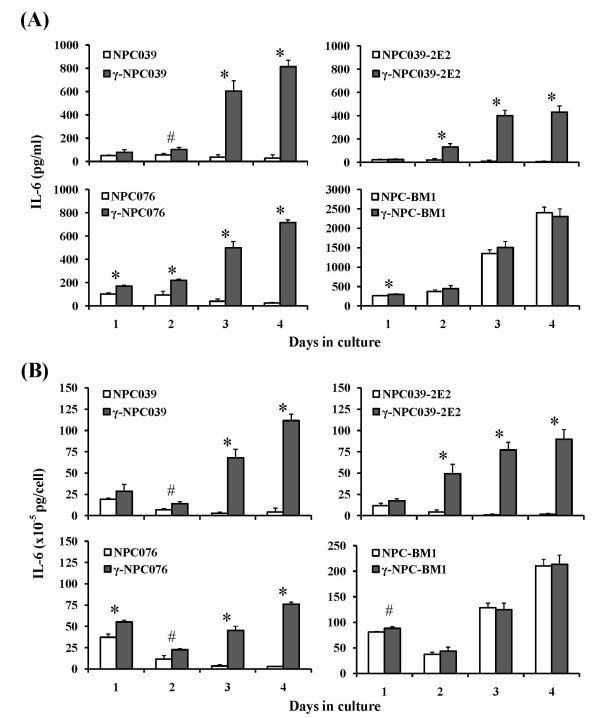
**Kinetics of IL-6 secretion in the spent media by nontransduced and *IFN-γ*-transduced NPC cell lines in culture**. Note that NPC039, NPC039-2E2 and NPC076 were derived from primary tumor lesions, while NPC-BM1 was derived from a bone marrow metastatic lesion. NPC039-2E2 was a clonal subline isolated from the NPC039 cell line. Significant levels of differences in IL-6 secretion between nontransduced and *IFN-γ*-transduced NPC cell lines are indicated (# *p *< 0.05, * *p *< 0.01).

We next examined the release of IL-6 in the spent media from 6 cultured P-NPC cell lines in a time course study. Low but detectable levels of IL-6 were secreted from all P-NPC cell lines with a maximal secretion at day 1 [(11.7 ~ 37.2) × 10^-5 ^pg/cell], which were thereafter diminished gradually to nearly undetectable down to day 4. By contrast, in *IFN-γ*-transduced cell counterparts, higher levels of IL-6 were released progressively peaking at day 3 or 4 [(76.1 ~ 111.6) × 10^-5 ^pg/cell]. When the patterns of IL-6 released in nontransduced and transduced cells of BM-NPCs were compared, results were very different from those in P-NPCs, in that gradual increases in IL-6 secretion up to 210.5 × 10^-5 ^pg/cell were detected as a function of time in nontransduced BM-NPCs, peaking at day 4. Moreover, the kinetic profile of IL-6 release was essentially unchanged following *IFN*-*γ* transduction (Figure 5). Similar results were obtained with NPC-BM3 cells of a short-term culture derived from a bone marrow metastatic lesion of a different NPC patient (results not shown).

**Figure 6:**
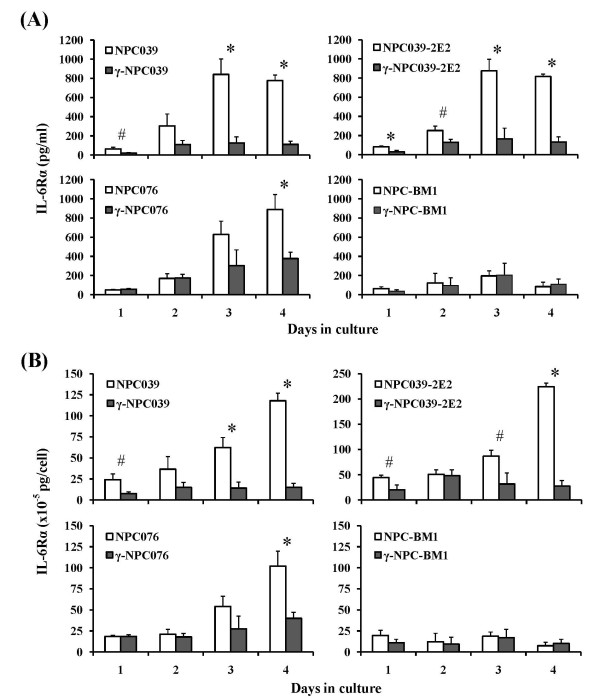
**Kinetics of IL-6Rα secretion in the spent media of cultured nontransduced and *IFN-γ*-transduced NPC cell lines**. Note that NPC039, NPC039-2E2 and NPC076 were derived from primary lesions, while NPC-BM1 was derived from a bone marrow metastatic lesion. NPC039-2E2 was a clonal subline isolated from the NPC039 cell line. Significant levels of differences in IL-6Rα secretion between nontransduced and *IFN-γ*-transduced NPC cell lines are indicated (# *p *< 0.05, * *p *< 0.01).

Kinetic experiments for IL-6Rα were also carried out with the spent media of the same panel of cultured NPC cell lines/sublines. As illustrated in Figure 6, progressively increased levels of soluble IL-6Rα were detected in culture supernatants of nontransduced P-NPCs with maximal levels of (101.9 ~ 224.3) × 10^-5 ^pg/cell at day 4. However, low levels of IL-6Rα [(15.2 ~ 40.0) × 10^-5 ^pg/cell] were detected in *IFN-γ*-transduced counterparts throughout the 4 days culture period. These results suggest an association of general suppression of IL-6Rα production with *IFN-γ*-transduction in P-NPCs.

### IL-6 secretion patterns in *IFN*-*γ*-transduced and nontransduced NPC clonal sublines

There was a possibility that the inducibility of IL-6 and suppression of IL-6Rα by *IFN-γ*-transduced NPC cells derived from primary lesions demonstrated in the previous experiments could be due to the selection of potentially high inducible subpopulations from the uncloned population of a given P-NPC cell line. To rule out this possibility, kinetic studies with four additional isolated clonal sublines NPC039-2E2, -6A1 and -7B2 were conducted. The different fluctuation patterns in IL-6 and IL-6Rα productions by nontransduced and *IFN-γ*-transduced cells are consistent among P-NPC cell lines and clonal sublines derived thereof (Figures 5 and 6), in which results of one such clonal subline NPC039-2E2 are presented. These results and the finding of IL-6 and IL-6Rα secretion patterns in NPC cell lines transduced with a vector of neogene without containing the *IFN-γ *gene resembled the baseline profiles exhibited by the nontransduced NPC cells (data not shown) exclude the possibility of transduction-mediated selection for high IL-6 producing or low IL-6Rα producing subpopulations.

## Discussion

The identification of an expanding role for IFN-γ in the phenotypic changes favouring for improved immunogenicity of neoplastic cells prompted us to search for soluble or cell bound molecules associated with NPC malignant cells, in addition to certain important molecules required for immune recognition, such as ICAM-1, HLA class I and HLA class II already documented in many cancer systems. In this study, we found that cultured BM-NPCs were able to constitutively produce appreciable protein levels of IFN-γ and IL-6, while cultured P-NPCs were not. Enhanced production of IFN-γ and IL-6 were markedly demonstrated in P-NPCs after they were transduced with the *IFN-γ *gene, but the magnitude of enhancing effects was much less in BM-NPCs. The most significant finding in this current study is the differential patterns of constitutive and response profiles of IL-6 and IL-6Rα following transduction with the *IFN-γ *gene, which clearly discriminate between cultured P-NPCs and BM-NPCs. Since only a limited number of NPC cell lines from distant metastatic sites other than bone marrow are currently available, we have not yet tested them in this study. Therefore, whether the similar differential modulation profile of BM-NPCs to *IFN-γ *gene-transduction could also be observed with other distant metastatic NPC cell lines remains to be determined.

The results of IL-6 and IL-6Rα secretion patterns in NPC cell lines transduced with a vector of neogene without containing the *IFN-γ *gene were essentially similar to those of the baseline profiles exhibited by the nontransduced NPC cells. In separate experiments in which P-NPCs and BM-NPCs were incubated independently with recombinant human IFN-γ at the concentrations of 100 ~ 600 U/ml for 48 h, we have obtained similar results of fluctuation in IL-6/IL-6Rα secretion as those with *IFN-γ *transduction (data not shown). These two lines of evidence indicate that the gene transduction process itself was not the cause of the changes in IL-6 and IL-6Rα secretion and that the observed effects were indeed as the result of the expression of the *IFN-γ *gene.

It has been documented that IL-6, a 184-amino acid protein with a molecular weight of 20.3 kDa was originally described by Hirano *et al. *[23], is a key cytokine required for plasma cell induction, B cell growth and antibody secretion, and activation of CD4 cells, thus favouring for Th2 immune responses [24]. The release of and response to IL-6 by myeloma cells [25], melanoma cells [26], and carcinoma cells of the head and neck [27], the breast [28] and the kidney [29] have been reported. The role of IL-6 release into the tumor microenvironment by malignant cells appears to vary and has been interpreted differently depending on tumor systems: from as the indicator of poorer prognosis, involvement in autocrine stimulatory growth, enhanced invasiveness, to representation of Th1 and/or Th2 immune responses [30]. In an autocrine system, IL-6 was known to regulate tumor cells proliferation and secretion of metalloproteinases, which are involved in tumor invasion and metastasis [31]. From the therapeutic standpoint, there were two studies that examined the role of IL-6 and its receptors in promoting antitumor responses. Porgador *et al*. [32] reported that the *IL-6 *gene transfection into Lewis lung carcinoma cells resulted in suppression of malignant phenotype and concomitant endowment with immunotherapeutic competence against parental tumor cells, signifying induction of Th1 immune response. Mackiewicz *et al*. [33] investigated into the possible role of IL-6 and its receptors in activating T cell immunity against melanoma. IL-6 exerted its activity through a membrane-bound receptor complex consisting of α (gp80) and β (gp130) subunits of the IL-6 receptor. In other instances, the soluble IL-6Rα (sIL-6Rα) subunit could often present itself as a soluble molecule, to form many IL-6/sIL6Rα complexes first, which are in turn attracted to two gp130 molecules (homodimers) expressed on most of the key immune effector cells including CD4^+^, CD8^+ ^(CTLs), antigen presenting cells, and NK cells. These interactions could lead to signal transduction of immune effector cells. The proposed mechanism of IL-6/sIL-6Rα complex is to activate the various immune cells indicated *via *IL-6Rβ, which in combination would lead to potent anti-tumor effects (Th1 immune responses) [34]. In other words, IL-6 complexed with sIL-6Rα has demonstrated qualitatively different biological activity from IL-6 alone in stimulating long-lasting anti-tumor immunity. Currently, we have been making efforts to develop a sensitive and reliable assay for soluble IL-6/IL-6Rα complex in order to study the relationship between IL-6, IL-6Rα and IL-6/IL-6Rα complex expressed and released by cultured NPC cells of primary and metastatic tumor origins under *IFN-γ*-transduced and nontransduced conditions.

IL-6 itself has been shown to exhibit the upregulation of HLA class I molecules and certain tumor-associated antigens such as carcinoembryonic antigen (CEA) in colon carcinoma cells, as does IFN-γ [35,36]. Further, IL-6 could synergize the effect of IFN-γ on the upregulation of HLA class I and CEA when both cytokines were used in combination at concentrations 20- to 100-fold less than that used either cytokine alone. Additionally, treatment of colorectal tumor cells with IL-6 potentiates the IFN-γ induction of HLA class II expression. We also demonstrated in NPC039 cells that IL-6 could upregulate HLA class I expression and that combined use of IL-6 and IFN-γ, each at a lower concentration (about 25- or 57-fold less than when IL-6 or IFN-γ was used alone) showed synergistic effects on enhanced expression of surface HLA class I molecules as compared with a single cytokine alone (Lee M-H and Liao S-K, unpublished). A similar observation has also been made very recently in a canine cell system [37]. It is therefore reasonable to assume that increased expression of HLA class I and HLA class II observed in cultured NPC cells derived from primary tumors after *IFN-γ *transduction must have been due to the combination effects of both IFN-γ and IL-6. These results also suggest that secretion of both IFN-γ and IL-6 which can be induced by the *IFN-γ *gene transduction in cultured P-NPCs should theoretically be in favour for enhanced tumor immunogenicity, when these genetically modified tumor cells are irradiated and administered subcutaneously in the autologous patients as tumor vaccines. Further studies are required to determine which one between cultured P-NPCs and cultured BM-NPCs could be the ideal choice of material for the preparation of NPC tumor vaccines. It is also important to recognize that given the expression of new potential therapeutic targets (T cell epitopes) that may appear in bone marrow metastatic cells, we may have to manipulate metastatic tumor cells differently from those derived from primary lesions in order to make both genetically modified tumor vaccine preparations to be therapeutically effective.

It is of interest to determine the precise mechanism by which *IL-6 *gene is activated or unaltered after *IFN-γ *transduction in cultured NPC cells from primary tumors or in cultured NPC cells from bone marrow metastatic lesions, respectively. It has been noted that in the early stage of NPC pathogenesis, activation of IL-6 production in epithelial cells of the nasopharynx is mediated by EBV-encoded late membrane protein-1 (LMP-1) and CD40 through the induction of NF-κB pathway [38]. The role of EBV in the pathogenesis of NPC has been thoroughly reviewed [39]. Since in nearly all primary tumors in either short-term or long-term cultures, relatively low levels of IL-6 could be detected in the spent media, despite the fact that no EBV DNA or EBV-encoded RNA (*EBER*) could be detected after a few *in vitro *passages (data not shown). This is consistent with the previous findings by others [40], suggesting that in contrast to EBV-infected B cells in which EBV infection persistently takes place in cultured B cells, an *in vitro *environment invariably excludes the EBV genome from epithelial cells. Nevertheless, the mechanism underlying the loss of EBV genome in culture remains illusive. It is possible that after the *IL-6 *gene was activated in EBV-infected epithelial cells of the primary site *in vivo*, this cytokine released by these epithelial cells even at such lower concentrations appears to be required for the maintenance of their malignant states *in vitro*. Moreover, it is tempting to speculate that to establish a micrometastasis in the bone marrow, only tumor cells secreting high levels of IL-6 within the primary tumor cell population may manage to survive in the new microenvironment, i.e., bone marrow, which is so different from the primary site. Alternatively, among primary tumor cells able to produce relatively low amounts of IL-6, only few cells metastasize hematogenously into bone marrow cavities where the *IL-6 *gene is reactivated to produce a high level of IL-6 in order to survive successfully. These two possibilities, which may not be mutually exclusive, have yet to be investigated. In light of the importance of tumor microenvironment in carcinogenesis and tumor progression, more attention has recently been paid to the role of bone marrow microenvironment in tumor dissemination [41]. Tumor cells secreting high levels of IL-6 and MIP-1α were found to be recruited to the bone marrow, where the balance between osteoblasts and osteoclasts was altered, leading to a series of changes in the endosteal and vascular niches. Further, bone marrow mesenchymal stem cells (MSC) tend to secrete a high level of IL-6 [41-43], which is known to play a pivotal role in the marrow space for further tumor metastatic dissemination [43].

Both IFN-γ and IL-6 share some key factors in their signal transduction pathways, which involve JAK tyrosine kinases and STATs (signal transducer and activator of transcription) [44]. Larner *et al*. [45] suggested that cytokines such as IFN-γ activated the DNA-binding proteins whose tyrosine residues were phosphorylated, and thus could modulate gene expression through activation of putative transcription factors by tyrosine-phosphorylation. The JAK-family kinases induce tyrosine phosphorylation of cellular transcription factors STATs, as well as their translocation into nucleus, thereby activating *IL-6 *gene [44-47]. Two alternate models for the requirement of two JAK kinases in the IFN signaling can be envisioned. Either kinase functions sequentially in a cascade, or alternatively they can function together as heterodimers to activate the downstream signaling pathways. Activated JAKs (e.g. JAK1 and JAK3) efficiently phosphorylate the autophosphorylation sites of both kinases, thus also favouring for the cross-activation model. Our results indicate clearly that *IFN-γ *transduction could merely stimulate P-NPC cells constitutively secreting low basal levels of IL-6 to produce much higher levels of IL-6, but could not stimulate BM-NPC cells that have constitutively been able to secrete this cytokine and IFN-γ in higher levels, to further release additional IL-6. This discrepancy is very likely to involve the differences in the utility of factors, such as JAK1, JAK2, JAK3, Tyk2, NF-κB, IFN regulatory factor 1 (IRF-1) and/or STATs (e.g. STAT-1 activated by IFN-γ; STAT-3 activated by IL-6), thereby creating a positive and a negative regulation for P-NPC and BM-NPC cells respectively [47]. In other words, the mechanism underlying the relative resistance of BM-NPC cells to be enhanced to secrete IL-6 in a much higher magnitude like P-NPC cells is possibly due to the insensitivity to the ongoing cooperation of IRF-1 and the p65 subunit of NK-κB for IL-6 activation at the level of IL-6 promoter by IFN-γ [45]. In this context, recent reports on the newly identified role of IL-6 in epithelial cancer, such as breast and lung carcinomas, are intriguing in that IL-6 has been implicated in tumorigenesis *via *the IL-6 receptor-activation-mediated phosphorylation of STAT3 [48,49] and that release of IL-6 results in a positive feedback loop causing further IL-6 upregulation and secretion. Following binding of this cytokine and its receptor, IL-6Rβ, causes upregulation of Notch-3 ligand Jagged 1, which triggers the upregulation of carbonic anhydrase IX (CA-IX), such changes leading to promote malignant features of cancer stem cells [28]. These results also suggest that the IL-6 and IFN-γ pathways in a background of genetic instability be involved in the acquisition of metastatic behaviour in BM-NPCs. This may be one of the reasons why the approach of *IFN-γ*-modified tumor cells has gradually been phased out from the list of cancer vaccines over the past decade. Finally, one should bear in mind that the role of EBV is not evoked in the present study with the use of the NPC cell lines investigated.

## Conclusion

Our present study has demonstrated that in NPC asides from the difference in drug sensitivity to G418 and expression of surface HLA class II (Table 1), the baseline and response profiles to *IFN-γ *transduction of IL-6 and IL-6Rα are also found to be different between cultured P-NPCs and BM-NPCs. Our results stress the importance to evaluate the endogenous levels of IL-6 and IL-6Rα in each NPC individually, if cultured NPC cells of the given tumor are to be considered for making tumor vaccines. Finally, the precise mechanism behind the activation of IL-6 and concomitant suppression of IL-6Rα expression in P-NPCs following *IFN-γ *transduction and why such a phenomenon in P-NPCs is so different from that of BM-NPCs are still the issues as yet to be explored. This resulting information is important in our better understanding of NPC biology, specifically, the role of IL-6 secretion in NPC progression and bone marrow micrometastasis.

## Competing interests

The authors declare that they have no competing interests.

## Authors' contributions

AS-BC, H-YW and H-CC performed the study and drafted the manuscript. C-KC, and M-HT participated in the design of this study, S-KL conceived, overall coordination of this study, and the interpretation of the results. All authors read and approved the final manuscript.

## Pre-publication history

The pre-publication history for this paper can be accessed here:

http://www.biomedcentral.com/1471-2407/9/169/prepub
